# Ecopsychiatry - when we are confronted as professionals with the need to respond to climate change

**DOI:** 10.1192/j.eurpsy.2026.10385

**Published:** 2026-07-31

**Authors:** S. Murawiec

**Affiliations:** Polish Psychiatric Association, Warsaw, Poland

## Abstract

**Abstract:**

Climate change constitutes an increasingly important context for understanding contemporary mental health problems. Its impact on mental health can be conceptualized across several interrelated domains.

**Gradual climate and environmental changes**, including rising temperatures, biodiversity loss, and transformation of familiar landscapes, are associated with experiences described as solastalgia, eco-anxiety, climate depression, ecological grief, and broader environmental distress, commonly linked to anxiety, depressive symptoms, hopelessness, and a sense of loss.

**Acute climate-related natural disasters**, such as floods, heatwaves, and wildfires, produce both immediate and long-term psychological consequences, including trauma-related disorders, and persistent disruptions of social functioning and community cohesion.

**Human-driven environmental degradation**, including deforestation and ecosystem destruction, often generates anger, sense of helplessness, and moral distress, particularly when individuals and local communities feel powerless in the face of intentional environmental harm.

At the interface of psychiatry and the natural environment, **ecotherapeutic and nature-based interventions** (forest therapy, therapeutic ornithology) support mental health through structured contact with natural environments, contributing to emotional regulation, stress reduction, improved mood, and cognitive functioning. A study published in Poland in 2025 indicated that approximately **60% of psychiatrists consider nature-based interventions to be beneficial for mental health**, both in clinical and non-clinical populations.

**Disclosure of Interest:**

None Declared

